# Ginsenoside Rb_1_ Enhances Plaque Stability and Inhibits Adventitial Vasa Vasorum via the Modulation of miR-33 and PEDF

**DOI:** 10.3389/fcvm.2021.654670

**Published:** 2021-05-28

**Authors:** Xiaoyan Yang, Lei Wang, Zihao Zhang, Jiayi Hu, Xiaoling Liu, Hao Wen, Minghao Liu, Xue Zhang, Hongyan Dai, Mei Ni, Rui Li, Rong Guo, Lei Zhang, Xiaorong Luan, Huili Lin, Mei Dong, Huixia Lu

**Affiliations:** ^1^The Key Laboratory of Cardiovascular Remodeling and Function Research, Chinese Ministry of Education, Chinese National Health Commission and Chinese Academy of Medical Sciences, The State and Shandong Province Joint Key Laboratory of Translational Cardiovascular Medicine, Department of Cardiology, Qilu Hospital, Cheeloo College of Medicine, Shandong University, Jinan, China; ^2^Department of Cardiology, Heart Center and Beijing Key Laboratory of Hypertension, Beijing Chaoyang Hospital Affiliated to Capital Medical University, Beijing, China; ^3^The Second School of Clinical Medicine, Binzhou Medical University, Yantai, China; ^4^State Key Laboratory of Cardiovascular Disease, Fuwai Hospital, National Center for Cardiovascular Diseases, Chinese Academy of Medical Sciences and Peking Union Medical College, Beijing, China; ^5^Department of Cardiology, Qingdao Municipal Hospital, Qingdao, China; ^6^Department of Cardiology, China-Japan Friendship Hospital, Ministry of Health, Beijing, China; ^7^Department of Cardiology, The Second Affiliated Hospital of Fujian Medical University, Quanzhou, China

**Keywords:** ginsenoside Rb_1_, atherosclerosis, plaque stability, vasa vasorum, PEDF

## Abstract

**Background:** Atherosclerosis is closely associated with proliferation of the adventitial vasa vasorum, leading to the atherosclerotic plaque progression and vulnerability. In this report, we investigated the role of Ginsenoside Rb_1_ (Rb_1_) on atherosclerotic plaque stabilization and adventitial vasa vasorum (VV) along with the mechanisms involved.

**Methods and Results:** Apolipoprotein E-deficient (ApoE^−/−^) mice were fed with a high-fat diet for 20 weeks, and then Ginsenoside Rb_1_ (50 mg/kg/d, intraperitoneal) was given for 4 weeks. Rb_1_ treatment significantly inhibited adventitial VV proliferation, alleviated inflammation, decreased plaque burden, and stabilized atherosclerotic plaques in apoE^−/−^ mice. However, the beneficial effects of Rb_1_ on atherosclerotic lesion was attenuated by overexpression of miR-33. The analysis from atherosclerotic plaque revealed that Rb_1_ treatment could result in an induction of Pigment epithelium-derived factor (PEDF) expression and reduction of the miR-33 generation. Overexpression of miR-33 significantly reverted the Rb_1_-mediated elevation of PEDF and anti-angiogenic effect.

**Conclusions:** Ginsenoside Rb_1_ attenuates plaque growth and enhances plaque stability partially through inhibiting adventitial vasa vasorum proliferation and inflammation in apoE^−/−^ mice. The anti-angiogenic and anti-inflammation effects of Rb_1_ are exerted via the modulation of miR-33 and its target gene PEDF.

## Introduction

Atherosclerosis (AS), a chronic and progressive disease characterized by structural and functional changes in the vascular wall, is becoming a major health burden worldwide (1). Adventitial vasa vasorum (VV) is considered to be an essential component of extracranial vessels which are integral for nutrients delivery and wastes elimination (2). Proliferation of the adventitial vasa vasorum is responsible for atherosclerotic lesion progression and vulnerability, which is closely associated with a high risk of plaque rupture (3, 4). Studies aimed to reduce VV by using angiogenesis inhibitors reveal therapeutic potential for anti-angiogenic intervention in treating atherosclerosis (4).

Ginseng, a traditional herbal medicine, has been used to treat various diseases over thousands of years. More and more researchers confirmed its biological activities in diverse pathological processes, including atherosclerosis, neurodegenerative diseases, diabetes, cancer, etc. (5, 6). Ginsenoside Rb_1_ is a representative component of ginseng which exhibits anti-angiogenesis, anti-obesity, anti-oxidative stress, anti-fatigue properties (5, 7). We have proved Rb_1_ can inhibit angiogenesis by modulating pigment epithelium-derived factor (PEDF) in HUVECs (7, 8), however, it remains unclear whether Rb_1_ has the ability to suppress vasa vasorum proliferation in atherosclerotic lesions and furthermore to stabilize AS plaque.

Pigment epithelial-derived factor (PEDF), a member of the serpin superfamily that acts as an endogenous inhibitor of angiogenesis and protector of eyes (9). Numerous studies in various models have shown anti-angiogenic effects that PEDF has on tumors, ocular diseases and cardiovascular diseases (10, 11). PEDF is a potent inhibitor of angiogenesis through pro-apoptotic effects on endothelial cells. It can also inhibit endothelial cell migration, endothelial tube formation, vessel sprouting and intratumoral neovascularization, and can decrease the levels of pro-angiogenic factors (11–13). Moreover, PEDF is considered to be protective against atherosclerosis because of its anti-oxidative, anti-inflammatory, anti-thrombogenic properties (14, 15), however, the anti-angiogenic effect and mechanisms in atherosclerosis remains unclear.

MicroRNAs (miRNAs) are 19–25-nt-long regulatory RNAs expressed in plants and metazoan animals (16). miRNAs regulate gene expression post-transcriptionally by base-pairing to target mRNAs. To date, miRNAs have been primarily identified as negative regulators of expression of cellular mRNAs, leading to translational repression and gene silencing. Increasing evidence show that miRNAs are involved in onset and progression of several human disorders such as infectious and immune non-infectious diseases, cancers, metabolic, and cardiovascular disorders (17). Moreover, miRNAs are involved in every stage of biological process of atherosclerosis and exhibit an important role in modulating the evolution of atherosclerotic plaque toward vulnerability and rupture (18, 19). However, it remains to be elucidated whether and which miRNAs are involved in Rb_1_-induced PEDF expression and anti-angiogenesis.

Here, we sought to explore whether ginsenoside Rb_1_ can suppress the proliferation of vasa vasorum and stabilize atherosclerotic plaque along with the possible mechanisms.

## Materials and Methods

### Animals

In total, 45 female apoE^−/−^ mice on a C57BL/6 background (8 weeks old, male) were obtained from Beijing University Animal Research Center (Beijing, China) and all mice were kept on a 12 h light/12 h dark cycle. All mice were fed with a high-fat diet (0.25% cholesterol and 15% cocoa butter, Beijing Keao Xieli, China) for 20 weeks. The animals were fasted overnight, anesthetized with 0.8% Pentobarbital Sodium (i.p., 60 mg/kg). Blood was collected for lipid analysis from left ventricular apex. The mice were then euthanized by exsanguination under general anesthesia. After perfusion with 0.9% NaCl, heart and aortas were dissected from aortic arch to iliac bifurcation. Tissues were immediately stored in liquid nitrogen or fixed with 4% paraformaldehyde overnight. All animal studies were carried out in the Key Laboratory of Cardiovascular Remodeling and Function Research, Chinese Ministry of Education, Chinese National Health Commission and Chinese Academy of Medical Sciences, The State and Shandong Province Joint Key Laboratory of Translational Cardiovascular Medicine, Qilu Hospital of Shandong University.

### Study Protocol

All apoE^−/−^ mice were randomly divided into 3 groups (*n* = 15 per group). The control group was given equal amount of saline and empty lentivirus. The Rb_1_ group were injected empty lentivirus through tail vein and the next day started administering with intraperitoneal Rb_1_ 50 mg/kg daily continued for 4 weeks before sacrificed for further analysis. For the gain of function experiment, mice recieved miR33 oligonucleotides through tail vein injection of lentiviruses together with Rb_1_ treatment (50 mg/kg/d for 4 weeks). Rb_1_ treatment started the next day after lentivirus injection.

After 4 weeks' treatment, all mice were weighted by electronic balance (Shimadzu Corporation, Kyoto, Japan) and euthanized. The aorta and heart were harvested for further analysis. Blood samples were collected by cardiac puncture in the mice fasted overnight to measure the serum levels of total cholesterol and low-density lipoprotein cholesterol.

The animal experimental protocol was complied with the Animal Management Rules of the Chinese Ministry of Health (document no. 55, 2001) and was approved by the institutional ethics committee of Shandong University.

### Adventitial Vasa Vasorum (VV) Staining

VV in adventitia were identified by perfusion of biotinylated Lycopersicon Esculentum (Tomato) lectin as described before (20). In brief, mouse was perfused in order with 0.9% NaCl, 1% paraformaldehyde, and 0.5% glutaraldehyde, 1% FBS, 200 μg biotinylated tomato lectin in 1% FBS, 1% FBS, and finally 0.9% NaCl for 3 min each. The whole aorta was carefully dissected and fixed in methanol at 4°C overnight. After being embedded in OCT, cryosections in 5 μm thickness were collected from the aortic root. Tomato lectin distribution was detected at the aortic root.

### Histology and Immunohistochemistry

After euthanasia, mice were perfused with saline and followed by 4% paraformaldehyde through cardiac apex. The heart and aorta were dissected and fixed in 4% paraformaldehyde overnight. The aortic root, obtained by separating heart from ascending aorta, was embedded in Optimal Cutting Temperature compound and sectioned for 5 μm thick. Serial cryosections, were selected every 100 μm and stained with hematoxylin and eosin (H&E) to locate the site of maximal plaque size. The en face aortas were stained with Oil-red O and quantified as lesion-area fraction. The remaining staining involved sections around the site of maximal plaque size. Lipid deposition and collagen content were identified by Oil-red O staining and Picrosirius Red staining, respectively. Areas positively stained for MOMA-2, α-smooth muscle actin, and inflammatory cytokines including IL-1β, IL-6, and TNF-α were also analyzed. Briefly, cryosections were brought to room temperature and washed by phosphate buffered saline (PBS). Slides were then immerged in 3% H_2_O_2_ for 10 min before washed by PBS. After blocking, targeted proteins were identified by specific antibodies (details see in Supplementary Table 1) mentioned above at 4°C overnight. The next day after incubation with HRP linked secondary antibodies, DAB reagent were used for color development. Sections reacted with non-immune IgG and secondary antibodies only were used as negative control. Positive area analysis was determined using Image-Pro Plus 6.0. The vulnerability index was calculated using the following equation: Vulnerability Index = (lipids%+MOMA-2%)/(SMCs%+collagens%) (21).

### Real Time PCR

For miRNA measurement, RNA from mice aorta was extracted by mirVana™ miRNA Isolation Kit according to the manufacturer's instructions (Applied Biosystems, USA). TaqMan™ MicroRNA Reverse Transcription Kit and TaqMan™ MicroRNA Assay were used for reverse transcription. Real-time PCR was performed using TaqMan™ Gene Expression Master Mix, MicroRNA Assay from Applied Biosystems and 7500 PCR machine (ABI). The negative control U6 was determined in parallel and used to normalize the relative expression of miR-33.

### Statistical Analysis

All data were presented as the mean ± SEM. Statistical analysis was carried out using one-way analysis-of-variance (one-way ANOVA) followed by LSD *post-hoc* test (SPSS Software, San Diego, CA, USA). Statistical comparisons between two groups were carried out by the student's *t*-test. *P* < 0.05 was considered significant.

## Results

### Rb_1_ Attenuated Atherosclerotic Plaque Growth and Enhanced Plaque Stability in apoE^–/–^ Mice

**Figure 1:**
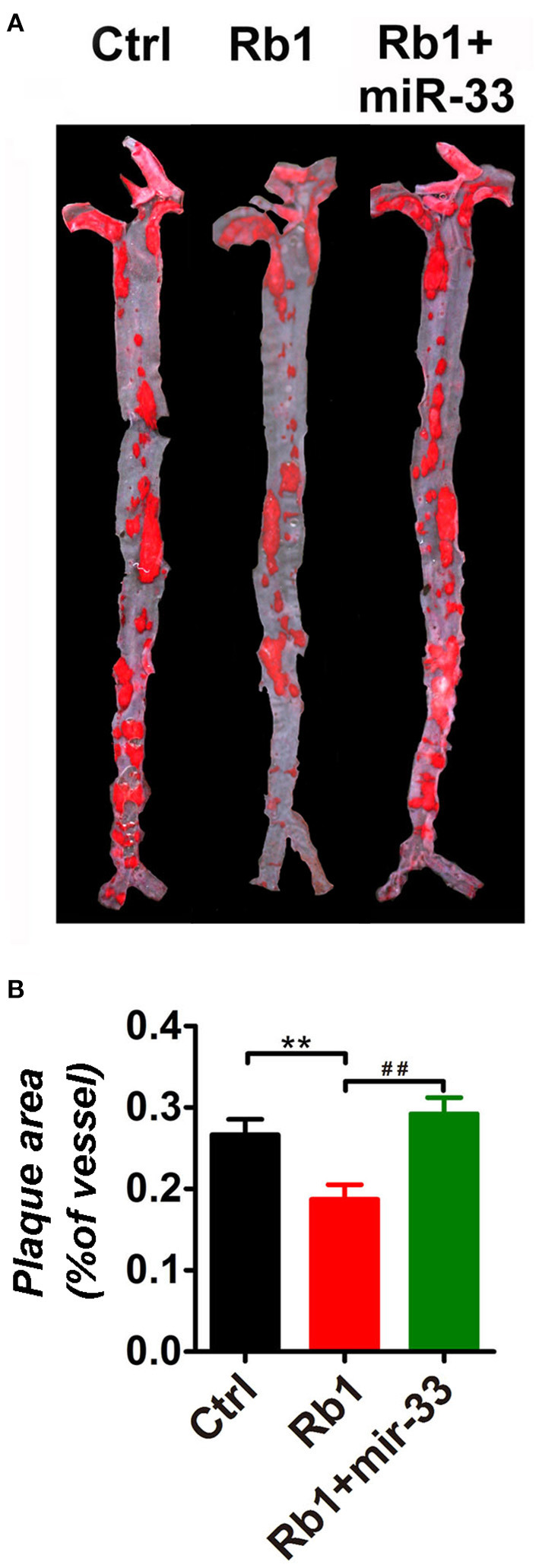
Rb_1_ attenuated atherosclerotic plaque growth in apoE^−/−^ mice. **(A)** General oil red O staining of en face atherosclerotic aorta stem of control group, Rb_1_-treated group as well as Rb_1_ + miR-33 treated apoE^−/−^ mice (*n* = 15/group). **(B)** Quantification of the ratio of plaque area vs. the total aorta stem area in control, Rb_1_-treated as well as Rb_1_ + miR-33 treated groups (*n* = 15/group). **p < 0.01; ##*p* < 0.01, one-way ANOVA.

**Figure 2:**
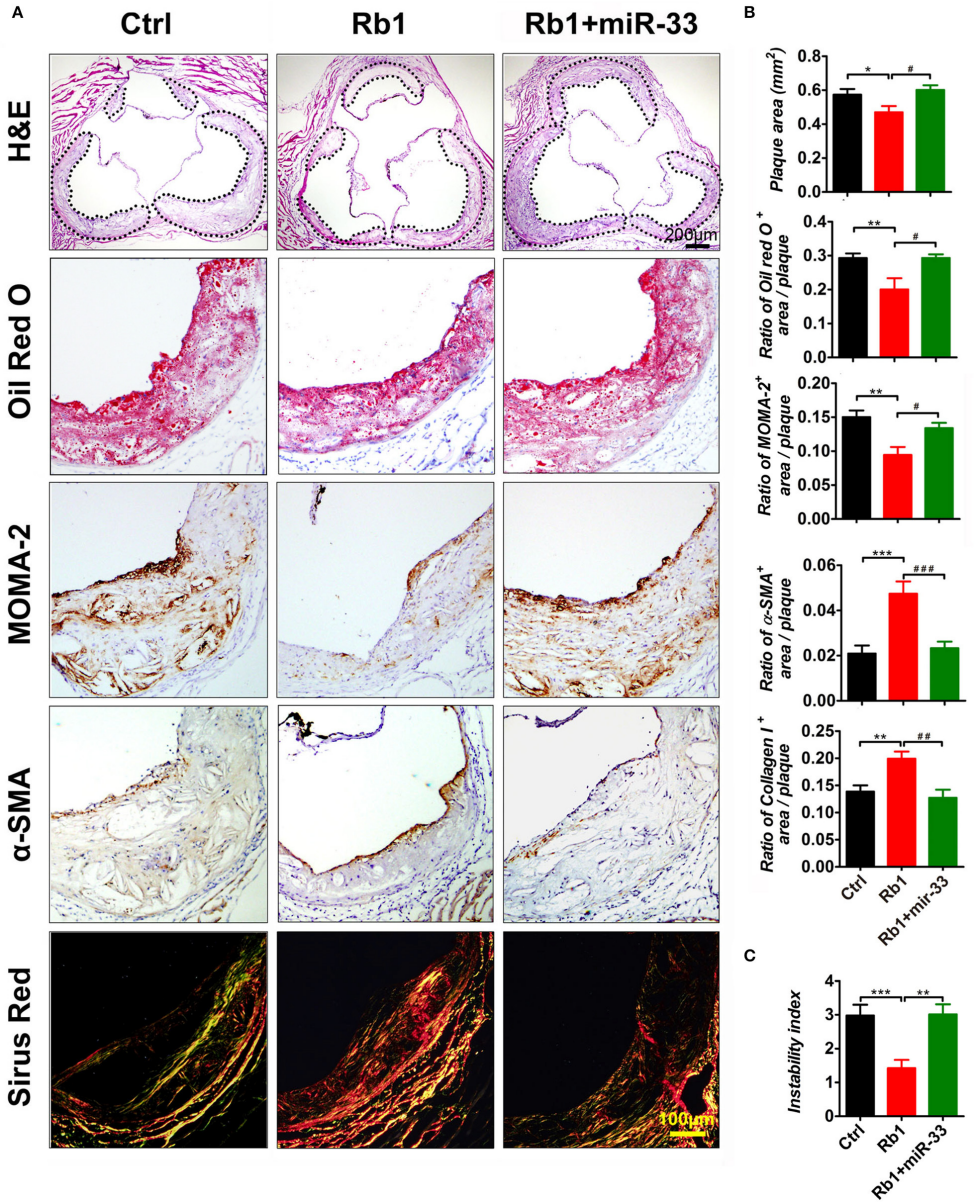
Rb_1_ attenuated atherosclerotic plaque growth and enhanced plaque stability in apoE^−/−^ mice. **(A)** Histological analysis of cross sectional aorta roots from three groups by staining with H&E, Oil Red O, anti-MOMA-2, anti-α-SMA, or Sirus Red. Dashed lines encircle atherosclerotic plaques (*n* = 14~15/group). **(B)** Quantification of the plaque area vs. the total cross sectional area of aorta root. Quantification of the ratio of Oil Red O positive area, MOMA-2 positive area, α-SMA positive area or the collagen I positive area vs. the total plaque area (*n* = 14~15/group). **(C)** Average values (+SEM) of atherosclerotic plaque instability index, which was calculated according to the formula: (oil red O^+^ area plus MOMA-2^+^ area)/(a-SMA^+^ area plus collagen I^+^ area) (*n* = 14~15/group). *p < 0.05; **p < 0.01; ***p < 0.001; #*p* < 0.05; ##*p* < 0.01; ###*p* < 0.001, one-way ANOVA.

As shown in Figure 1, there is a significant reduction of the relative en face atherosclerotic lesion area (Figure 1A) as well as the cross sectional lesion size at the aortic root (Figure 2A), which demonstrated that the extent of atherosclerosis was significantly decreased in Rb_1_ treated mice (Figures 1B, 2B). Furthermore, immunohistochemistry analysis also revealed that Rb_1_ treatment exerted notable effects on upregulating the contents of SMCs (Figure 2A) and collagen (Figure 2A). Conversely, Rb_1_ treatment could also reduce the accumulation of macrophages (Figure 2A) and lipid (Figure 2A). As a consequence, the plaque vulnerability index, which was calculated according to the formula: (oil red O^+^ area plus MOMA-2^+^ area)/(a-SMA^+^ area plus collagen I^+^ area), was dramatically decreased in Rb_1_-treated mice (Figure 2C). All the above results indicated that Rb_1_ treatment could enhance AS plaque stability.

### Rb_1_ Inhibited VV Proliferation in Atherosclerotic Plaques of apoE^–/–^ Mice

**Figure 3:**
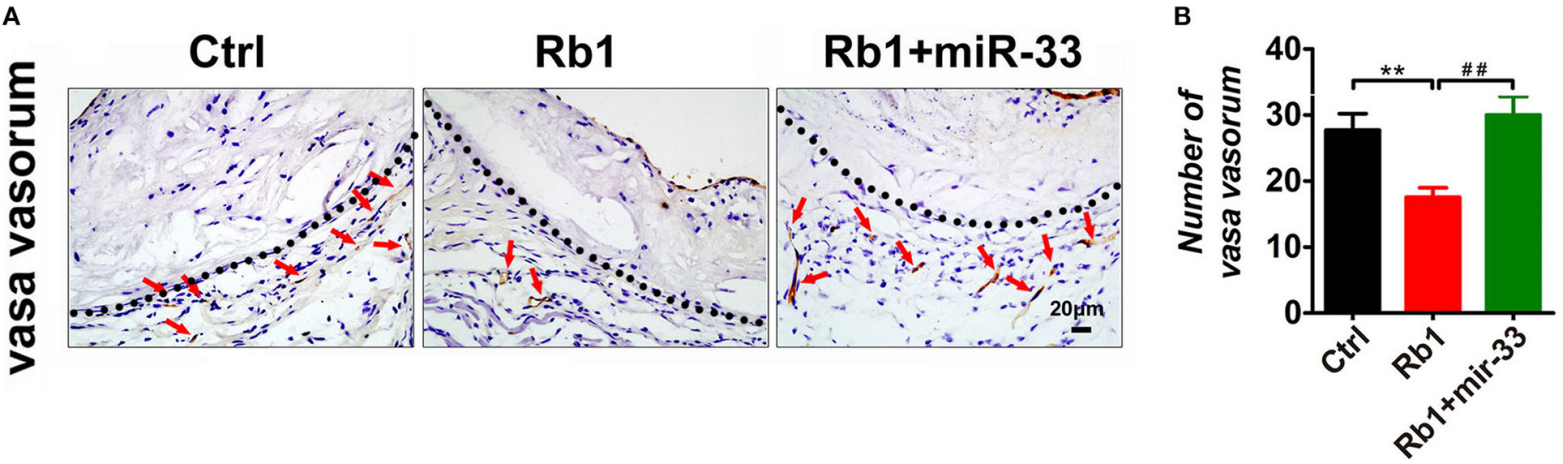
Analysis of VV expression in atherosclerotic plaques of apoE^−/−^ mice. **(A)** Histological analysis of adventitial VV proliferation at aorta roots of control group, Rb_1_-treated group as well as Rb_1_ + miR-33 treated apoE^−/−^ mice, by staining with Tomato lectin (*n* = 6/group). Tunica media and adventitia are separated by dotted lines. **(B)** Quantification of VV number in control group, Rb_1_-treated group and Rb_1_ + miR-33 treated apoE^−/−^ mice (*n* = 6/group). **p < 0.01; ##*p* < 0.01, one-way ANOVA.

Recent evidence has strongly suggested that atherosclerosis is an angiogenic disease. Angiogenesis has been suggested to significantly contribute to the aberrant neovascularization, which is thought to affect atherosclerotic plaque progression and destabilization (4, 22). Considering the anti-angiogenic role of Rb_1_, average number of vasa vasorum in adventitia surrounding the atherosclerosis plaques in aortic roots were counted to uncover whether Rb_1_ could inhibit neovascularization in apoE^−/−^ mice. As shown in Figure 3A, adventitia, the outermost layer of blood vessel wall, was separated from tunica media by dotted lines. We found that Rb_1_-treated group had less vasa vasorum, indicating that Rb_1_ could inhibit proliferation of VV in plaques of apoE^−/−^ mice (Figures 3A,B).

### Rb_1_ Inhibited the Inflammation of Atherosclerotic Plaques of apoE^–/–^ Mice

**Figure 4:**
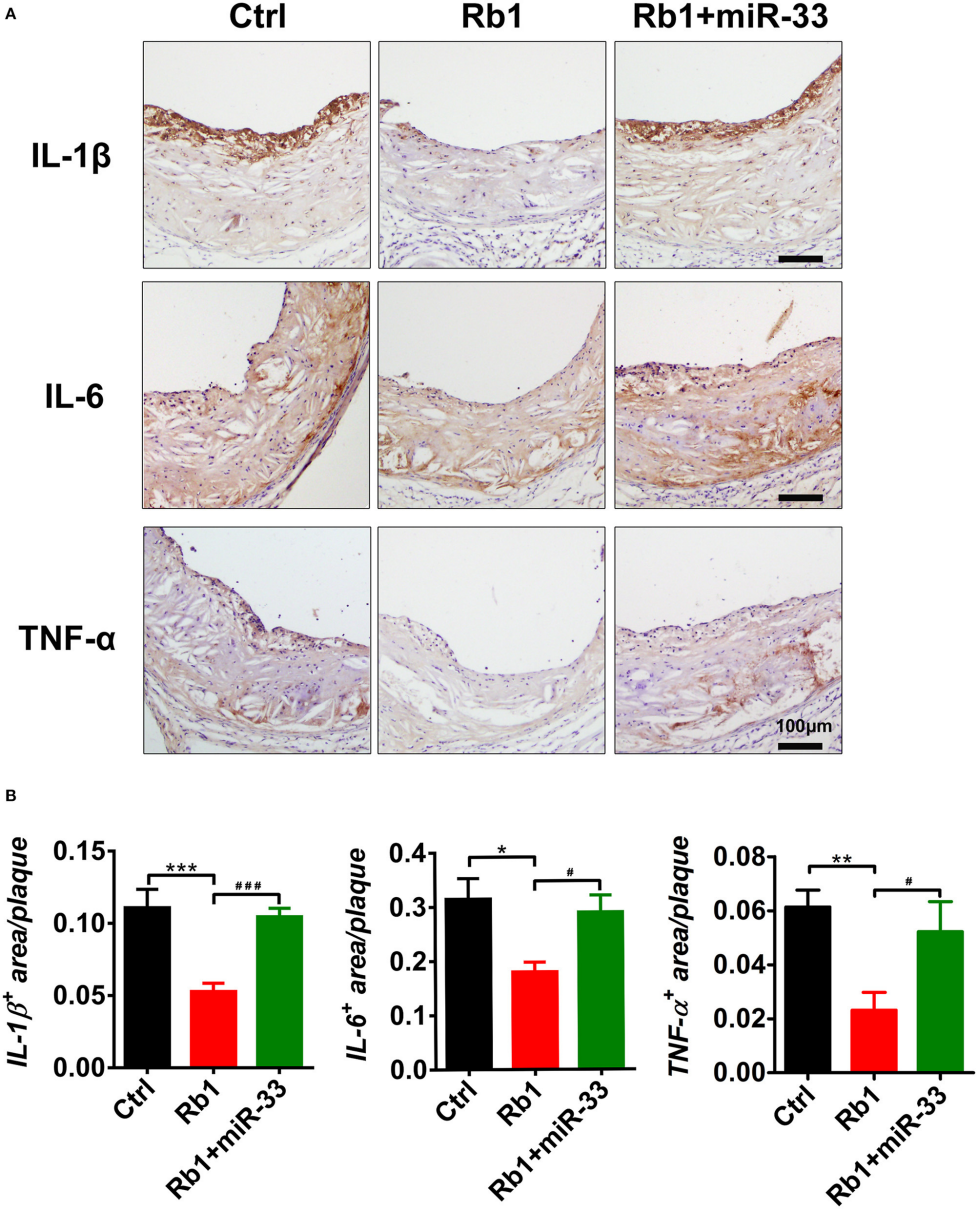
Analysis of inflammation-related cytokines in atherosclerotic plaques of three groups of apoE^−/−^ mice. **(A)** Immuno-histological analysis of cross sectional aorta roots of control group, Rb_1_-treated group as well as Rb_1_ + miR-33 treated apoE^−/−^ mice, by staining with anti-IL-1β, anti-IL-6, and anti-TNF-α (*n* = 10~15/group). **(B)** Quantification of inflammatory cytokines positive area vs. total cross sectional plaque areas of control group, Rb_1_-treated group as well as Rb_1_ + miR-33 treated apoE^−/−^ mice (*n* = 10~15/group). *p < 0.05; **p < 0.01; ***p < 0.001. #*p* < 0.05; ###*p* < 0.001, one-way ANOVA.

The immunohistochemical studies of inflammatory cytokines showed that, Rb_1_ treatment significantly reduced the IL-1β expression levels (Figure 4A). The statistical analysis showed compared with the control group, the Rb_1_ treated group tends to have lower expression level of IL-1β, and it has significant difference (Figure 4B). Similarly, the Rb_1_ treatment also significantly reduced the expression levels of IL-6 and TNF-α (Figures 4A,B). All these results indicated that Rb_1_ treatment could inhibit the AS plaque inflammation, which may hold back the development of AS plaque, as well as promote the plaque stabilization.

### The Beneficial Effects of Rb_1_ on Atherosclerosis Could Be Attenuated by miR-33 Overexpression

In this study, apoE^−/−^ mice transduced with lentiviruses expressing miR-33 were applied to study gain of function. miR-33 overexpression attenuated the beneficial effects of Rb_1_ on atherosclerosis. Compared with Rb_1_-treated group, miR-33 over expression significantly increased the accumulation of macrophages (Figure 2A) and lipid (Figure 2A), decreased the contents of SMCs (Figure 2A) and collagen (Figure 2A). Therefore, the plaque vulnerability index was dramatically increased in miR-33 overexpression mice (Figure 2B). Moreover, upregulation of miR-33 could significantly attenuate the anti-inflammation effects of Rb_1_ by decreasing the expression levels of IL-1β, IL-6, and TNF-α within AS plaques in apoE^−/−^ mice (Figures 4A,B). Figure 3 also showed that Rb_1_-mediated VV inhibition was abrogated by overexpression of miR-33. Together, these results demonstrated that miR-33 has profound negative impact on the Rb_1_-mediated enhancement of plaque stability, reduction of plaque burden, anti-inflammation and inhibition of VV in apoE^−/−^ mice.

### miR-33 Was Involved in Rb_1_-Induced PEDF Expression in as Plaque in apoE^–/–^ Mice

**Figure 5:**
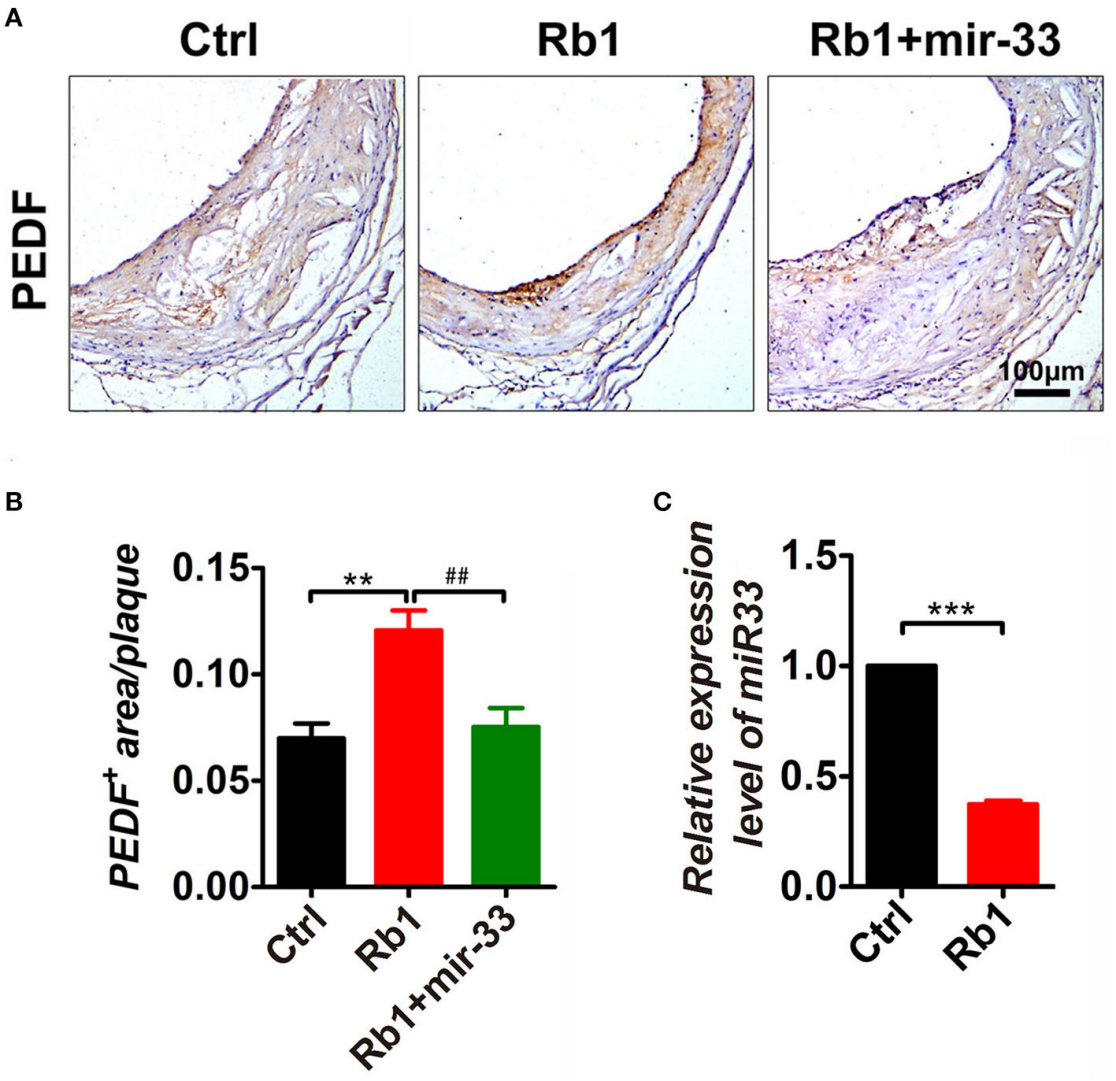
miR-33 was involved in Rb_1_-induced PEDF expression in AS plaque in apoE^−/−^ mice. **(A)** Immuno-histological analysis of cross sectional aorta roots of control group, Rb_1_-treated group as well as Rb_1_ + miR-33 treated apoE^−/−^ mice, by staining with anti-PEDF (*n* = 14~15/group). **(B)** Quantification of PEDF positive area vs. total cross sectional plaque areas of control group, Rb_1_-treated group as well as Rb_1_ + miR-33 treated apoE^−/−^ mice (*n* = 14~15/group), one-way ANOVA. **p < 0.01; ## *p* < 0.01. **(C)** The expression levels of miR-33 of control group and Rb_1_ treated group (*n* = 4/group). ***p < 0.001, student's t-test.

As shown in Figures 5A,B, Rb_1_ treatment led to the induction of PEDF expression in AS plaque. Interestingly, miR-33 expression was also significantly downregulated in Rb_1_ treated apoE^−/−^ mice by using real-time PCR (Figure 5C), which was consistent with the data in HUVECs published before (8). Overexpression of miR-33 results in a significant regression of Rb_1_-mediated PEDF elevation in AS plaque in apoE^−/−^ mice (Figures 5A,B).

## Discussion

In this study, we found that Rb_1_ treatment could inhibit VV proliferation, decrease plaque burden, suppress lesion inflammation and stabilize atherosclerotic plaques in apoE^−/−^ mice. However, the modulation of atherosclerosis progression by Rb_1_ was inhibited by overexpression of miR-33. Rb_1_ could decrease miR-33 level and increase PEDF expression in AS plaque. Overexpression of miR-33 significantly reverted the Rb_1_-mediated elevation of PEDF and anti-angiogenic effect. In summary, this study reveals that ginsenoside Rb_1_ could attenuate plaque growth and enhance plaque stability by inhibiting adventitial VV proliferation. Moreover, the anti-angiogenic effects of Rb_1_ are achieved via modulating miR-33 and its target gene PEDF.

Vasa vasorum is a specialized microvasculature that plays a major role in normal vessel wall biology and pathology (23). Under physiological conditions, the adventitial vasa vasorum takes up molecules that are transmitted from the blood to the adventitia by mass transport through the arterial wall (24). Lots of studies both in humans and in animal models demonstrate that increased vasa vasorum in the adventitia is associated with plaque instability and rupture, which can cause life-threatening cardiovascular events (22, 23, 25). Increased permeability of VV fuels atherosclerotic plaque progression. On the contrary, blockade of vasa vasorum angiogenesis not only attenuates early neointima formation in experimental hypercholesterolemia (26), but also reduces plaque growth in atherogenic female LDLR(-/-)ApoB-48-deficient mice (27). Thus, angiogenesis intervention may have beneficial effects on atherosclerotic plaque progression and stability.

Ginseng, one of the most well-known medical herbs, has a long history of use for managing various diseases in Asian countries (5). Ginsenosides are responsible for most pharmacological activity of ginseng and thus widely used to alleviate various diseases (28). Considerable attention has been focused on ginsenoside Rb_1_ for its beneficial effects on several types of diseases, including metabolic, vascular, and central nervous system diseases. It has been reported that ginsenoside Rb_1_ possesses anti-angiogenic potentiality in HUVECs (7). Although previous studies suggested the anti-atherogenic effects of Rb_1_ with other mechanisms (29, 30), lipid profiling revealed that Rb_1_ merely has a trend on lowering blood total cholesterol and low-density lipoprotein cholesterol, it is not clear whether ginsenoside Rb_1_ can take an effect in atherosclerosis based on its anti-angiogenic effect. In the present study, we firstly found Rb_1_ played an atheroprotective role through promoting atherosclerotic plaque stability, and this beneficial effect was partly achieved through the modulation of angiogenesis via upregulating PEDF level.

Pigment epithelium-derived factor (PEDF), first discovered in conditioned medium from retinal pigment epithelium (RPE) cell culture, is considered as the most potent endogenous inhibitor of angiogenesis known to date, which is 7 times than endostatin (9). PEDF is essential for maintenance of the avascular state of cornea. It has an ability to block abnormal neovessels without overt harm to the established retinal vessels even the high doses (31). In addition to the potent and selective anti-angiogenic function, PEDF also has neuroprotective, antioxidant, anti-inflammatory, and antithrombotic properties (10, 32). These pleiotropic characteristics of PEDF provide the advantages for the therapy of atherosclerotic diseases. Previous study had reported that PEDF play a protective role in blocking thrombus formation on a disrupted atherosclerotic plaque, leading to the inhibition of the atherosclerotic plaque rupture in the carotid artery (32). Additionally, it had been suggested local overexpression of PEDF within atherosclerotic lesion might block angiogenesis in these sites (9), contributing to the induction of plaque stability. An important finding in our present study was that Rb_1_ can upregulate PEDF expression in AS lesions, by which it exerted a protective effect on atherosclerotic plaque stability. Previously we showed that Rb_1_ had anti-angiogenic activity, even at the relatively low doses (5–20 nM). The anti-angiogenic effect of Rb_1_ was reverted by the neutralizing antibody to PEDF, which revealed that this action was exerted through activating PEDF (7). In HUVECs we found that Rb_1_ at the dose of 10 nM, could increase PEDF protein expression, without affecting PEDF mRNA level (8). Based on the inductive effect of Rb_1_ (10 nM) on PEDF protein level but not mRNA level, we propose that the post-transcriptional modulation may be involved in this process.

MicroRNAs (miRNAs), which are a broad group of endogenous small non-coding molecules, play a key role in post-transcriptional gene processes through inhibiting the translation or destabilizing of mRNA. Recently, the possible roles of microRNAs in ginsenosides-mediated angiogenesis came to our attention (33). We verified that Rb_1_ tampered the expression of miR-33 in atherosclerotic plaques of apoE^−/−^ mice, with the help of real-time PCR. Furthermore, miR-33 partially reverted the elevation of PEDF by Rb_1_. These data supported that Rb_1_ could promote PEDF protein expression via down-regulation of miR-33 *in vivo*. This is the first report that miR-33 plays a key role in Rb_1_-mediated PEDF induction. It has been approved by our team that PEDF is one of the target genes of miR-33. MiR-33 was shown to partially tamper the inhibitory effects of Rb_1_ on vasa vasorum proliferation in apoE^−/−^ mice. This research provides direct evidence of pro-angiogenic effect of miR-33 *in vivo*. This is an interesting finding, which would broaden the functional research of miR-33 in angiogenesis-related research and deepen our understanding of miR-33 regulatory mechanisms.

Inflammatory cytokines are well-known potent triggers of neovascularization in various settings like acute ischemic damage and tumor growth (2). Inflammation and VV formation tend to feed each other in a vicious cycle in that the plaque inflammation increases oxygen demand, thereby promoting neovascular growth. While neovascularization progresses, increased permeability of VV recruits more inflammatory cells into the plaque. In this study, we found that Rb_1_ treatment could significantly downregulate the expression levels of IL-1β, IL-6, and TNF-α in plaques along with the amelioration of the atherosclerotic plaques. While miR-33 could significantly reverse the beneficial anti-inflammation effects of Rb_1_ on the atherosclerotic plaques by up regulation the expression levels of IL-1β, IL-6, and TNF-α.

Given all that, miR-33 overexpression could decrease PEDF level in atherosclerotic plaque and therefore enhance the angiogenic activity, highlighting the key role of miR-33 in PEDF-mediated angiogenic pathway. And what's more, Rb_1_ could reduce miR-33 expression, resulting in the increment of PEDF protein. Consequently, we conclude that Rb_1_ exerts its anti-angiogenic action via regulation of miR-33 and its target gene PEDF.

In conclusion, a remarkable inhibition of VV proliferation, plaque burden reduction and atherosclerotic plaques stabilization were observed in Rb_1_ treated apoE^−/−^ mice, which was blocked by the overexpression of miR-33. The possible mechanisms behind Rb_1_-exerted anti-atherosclerotic function involved the downregulation of miR-33, leading to the stimulation of PEDF. Therefore, our finding provides evidence for a therapeutic potential of Rb_1_ in treating atherosclerotic disease related to pathological angiogenesis.

## Data Availability Statement

The raw data supporting the conclusions of this article will be made available by the authors, without undue reservation.

## Ethics Statement

The animal study was reviewed and approved by the institutional ethics committee of Shandong University.

## Author Contributions

MD and HLu generated ideas of this study, analyzed the results, and wrote the manuscript. XY, LW, and MD performed most experiments, analyzed the data, and help to wrote the manuscript. ZZ, JH, XLi, HW, ML, XZ, RL, and RG performed some experiments and analyzed the data. HD, MN, LZ, XLu, and HLi actively participated in discussions and supervised. XY, LW, and MD for performing the experiments. All authors contributed to the article and approved the submitted version.

## Conflict of Interest

The authors declare that the research was conducted in the absence of any commercial or financial relationships that could be construed as a potential conflict of interest.
