# Supplementary material for: Ginsenoside Rb1 Enhances Plaque Stability and Inhibits Adventitial Vasa Vasorum via the Modulation of miR-33 and PEDF
Source: Front Cardiovasc Med. 2021 May 28;8:654670. doi: 10.3389/fcvm.2021.654670 (PMC8192703; doi:10.3389/fcvm.2021.654670)
Supplement: Supplementary file 1 [file Data_Sheet_1.docx]

**SUPPLEMENTARY MATERIAL**

**Ginsenoside Rb1 enhances plaque stability and inhibits adventitial vasa vasorum via the modulation of miR-33 and PEDF**

Xiaoyan Yang^a,b,1^, Lei Wang^a,1^, Zihao Zhang^a^, Jiayi Hu^a^, Xiaoling Liu^a^, Hao Wen^a,c^, Minghao Liu^a,d^, Xue Zhang^a,e^, Hongyan Dai^e^, Mei Ni^a^, Rui Li^f^, Rong Guo^g^, Lei Zhang^a^, Xiaorong Luan^a^, Huili Lin^g^, Mei Dong^a^*, and Huixia Lu^a^*

^a^ The Key Laboratory of Cardiovascular Remodeling and Function Research, Chinese Ministry of Education, Chinese National Health Commission and Chinese Academy of Medical Sciences, The State and Shandong Province Joint Key Laboratory of Translational Cardiovascular Medicine, Qilu Hospital of Shandong University, 107 Wenhuaxi Road, 250012 Jinan, China

^b^ Heart Center and Beijing Key Laboratory of Hypertension, Beijing Chaoyang Hospital Affiliated to Capital Medical University, Beijing, 100020, China

^c^ Binzhou Medical University, Binzhou, 256603, China

^d^ State Key Laboratory of Cardiovascular Disease, Fuwai Hospital, National Center for Cardiovascular Diseases, Chinese Academy of Medical Sciences and Peking Union Medical College, Beijing, 100037, People’s Republic of China

^e^ Department of cardiology, Qingdao Municipal Hospital, Qingdao, 266011, China

^f^ Department of Cardiology, China-Japan Friendship Hospital, Ministry of Health, Beijing, 100029, China

^g^ Department of Cardiology, The Second Affiliated Hospital of Fujian Medical University, Quanzhou 362000, Fujian, People’s Republic of China

*Corresponding author: [meidong_sdu@163.com](mailto:meidong_sdu@163.com), [luhuixia@sdu.edu.cn](mailto:luhuixia@sdu.edu.cn)

^1^ These two authors contributed equally to this work.


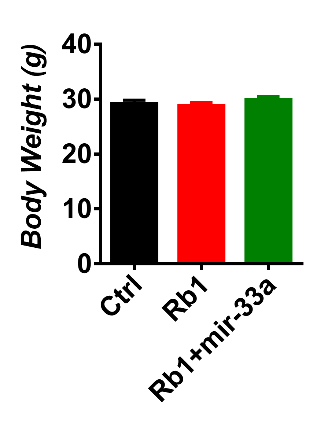


**Supplemental Figure 1**. Body weight of control group, Rb_1_-treated group as well as Rb_1_ + miR-33a treated apoE^-/-^ mice (n=15/group).


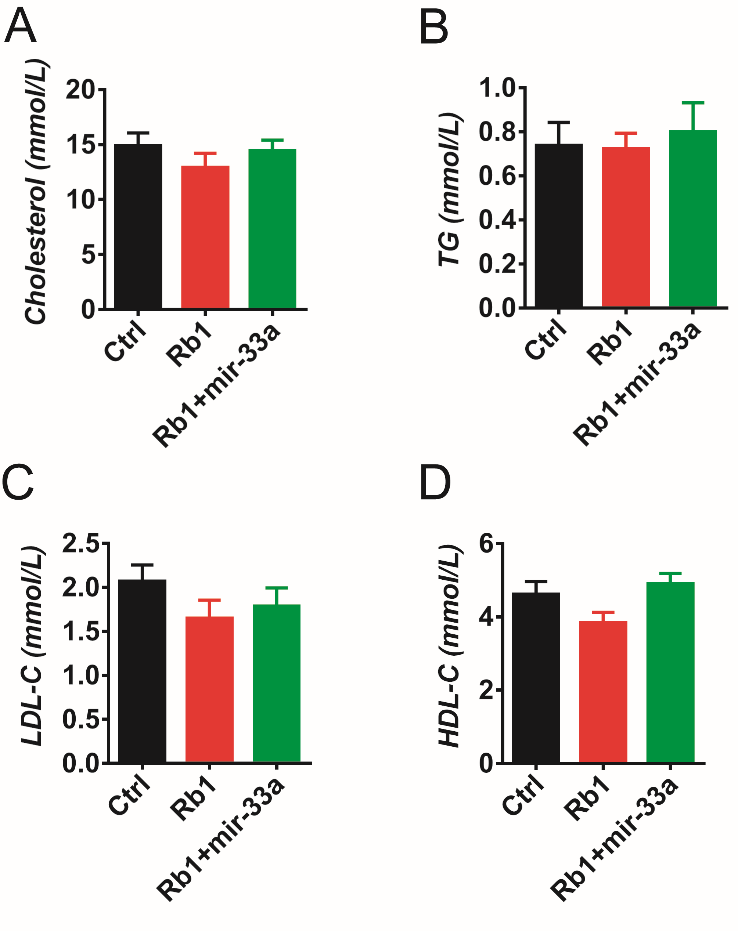


**Supplemental Figure 2**. Blood lipid profile of control group, Rb_1_-treated group as well as Rb_1_ + miR-33a treated apoE^-/-^ mice (n=15/group). (A). Total cholesterol, (B). Triglycerides (TG), (C). Low-density lipoprotein cholesterol (LDL-C), and (D). High-density lipoprotein cholesterol (HDL-C) levels from three groups.

**Supplemental Table 1**

| Material | Vendor or Source | Catalog # |
| --- | --- | --- |
| Ginsenoside Rb_1_ | Fleton Natural Products Co., Ltd | 41753-43-9 |
| MiR-33 lentivirus | Genechem |  |
| Control lentivirus | Genechem |  |
| Biotinylated Lycopersicon esculentum (Tomato) lectin | Vector Laboratories | B-1175 |
| Anti-MOMA-2 | AbD Serotec | MCA519 |
| Anti-α smooth muscle actin | Abcam | ab5694 |
| Anti-PEDF | abbiotec | 250819 |
| Oil red O | Sigma | O9755 |
| Picrosirius red | Sigma | 365548 |
| Anti-IL-1β | Abcam | ab33774 |
| Anti-IL-6 | Abcam | ab6672 |
| Anti-TNF-α | Abcam | ab66579 |
| mirVana™ miRNA Isolation Kit | Thermo Fisher Scientific | AM1561 |
| TaqMan™ MicroRNA Reverse Transcription Kit | Applied Biosystems | 4366596 |
| TaqMan™ Gene Expression Master Mix | Applied Biosystems | 4369016 |
| TaqMan™ MicroRNA Assay | Applied Biosystems | 4427975 |
